# Identification and Free Radical Scavenging Activity of Oligopeptides from Mixed-Distillate Fermented Baijiu Grains and Soy Sauce Residue

**DOI:** 10.3390/metabo14060298

**Published:** 2024-05-24

**Authors:** Yunhao Zhao, Xiangyue Liu, Sijie Zhang, Zhengwei Wang, Shanlin Tian, Qiang Wu

**Affiliations:** 1 College of Food and Chemical Engineering, Shaoyang University, Shaoyang 422000, China; 13769193967@163.com (Y.Z.); 19873971949@163.com (X.L.); 18150679603@163.com (S.Z.); wzw158789@163.com (Z.W.); palesilver2024@163.com (S.T.); 2 Hunan Key Laboratory of New Technology and Application of Ecological Brewing, Shaoyang 422000, China; 3 Shaoyang Engineering Technology Research Center of Functional Fertilizer, Shaoyang 422002, China

**Keywords:** sauce-aroma Baijiu, oligopeptide, identification, antioxidant activity, molecular docking

## Abstract

This study aimed to explore the potential antioxidant activity and mechanism of oligopeptides from sauce-aroma Baijiu. The oligopeptides of Val-Leu-Pro-Phe (VLPF), Pro-Leu-Phe (PLF), Val-Gly-Phe-Cys (VGFC), Leu-Tyr-Pro (LYP), Leu-Pro-Phe (LPF), and Phe-Thr-Phe (FTF) were identified by liquid chromatography–mass spectrometry (LC–MS) from the mixed-distillate of Baijiu fermented grains and soy sauce residue (MDFS). The antioxidant mechanism of these oligopeptides on scavenging DPPH•, ABTS•^+^, and hydroxide radicals was investigated, respectively. Among them, VGFC had the strongest potential antioxidant activity, which was responsible for its hydrogen bonds with these radicals with high affinity. The binding energies between VGFC and these radicals were −1.26 kcal/mol, −1.33 kcal/mol, and −1.93 kcal/mol, respectively. Additionally, free radicals prefer to bind the oligopeptide composed of hydrophobic amino acid residues such as Leu, Val, Phe, and Pro, thus being scavenged for exerting antioxidant activity. It provided a new idea for the development and utilization of bioactive oligopeptides in sauce-aroma Baijiu.

## 1. Introduction

In the process of metabolism, reactive oxygen species (ROS) are produced by the oxidation of mitochondrial respiratory chain peroxidase and oxidase. The level of ROS is controlled by the enzyme and non-enzyme antioxidant defense systems and maintained in a certain range [1]. When the body is in a normal physiological state, an appropriate amount of free radicals can participate in the intracellular signal cascade to resist pathogen invasion [2]. However, excessive free radicals alter normal ROS levels and lead to oxidative stress. Increased oxidative stress can lead to the development of diabetes, cardiovascular disease, and cancer [3]. Antioxidants can inactivate free radicals and prevent or terminate free radical chain reactions by hydrogen atom transfer, electron transfer, or chelation of metal ions [4]. Therefore, it is particularly important to find compounds with antioxidant activity that scavenge free radicals. Antioxidant peptides are considered to be safe and efficient natural antioxidants. Additionally, for the determination of the antioxidant activity of peptides, antioxidant capacity assays (e.g., DPPH, ABTS, etc.) are commonly used. However, the methods of ABTS and DPPH do not often follow the same mechanism as observed with peroxyl radicals [5], which makes them suitable only for preliminary screening.

Baijiu is a traditional fermented alcoholic beverage that originated in China and is usually obtained by the natural fermentation of grains (wheat, sorghum, corn, and rice). Alcohols, esters, aldehydes, and other flavor substances play an important role in shaping the unique taste and aroma of liquor [6]. However, affected by environmental factors and storage conditions, Baijiu flavor substances are easy to volatilize, resulting in quality decline and taste changes. To solve these problems, researchers can better retain flavor substances and reduce their volatilization by reducing wine storage temperature and optimizing fermentation, distillation, aging, and other brewing processes. The addition of antioxidants is also an effective method, which can reduce the oxidation degree of flavor substances, slow down the volatilization process, and maintain the stability of wine flavor [7]. In the past, antioxidants, such as sulfur dioxide, ascorbic acid, and sulfite, were mainly used in fermented alcoholic beverages [8,9,10]. However, excessive use of antioxidants leads to odor in wine [11] and safety risks such as allergic reactions, dermatitis, urticaria, angioedema, and allergic reactions [12]. Antioxidant oligopeptides are safe and efficient natural antioxidants such as glutathione (GSH) in wine quality, and aging is now widely accepted. GSH can prevent the harmful effects of oxidation on wine aroma during aging [13]. Moreover, Baijiu was also found to contain a variety of antioxidant oligopeptides. Arg-Asn-His (RNH) with anticancer and antioxidant activity was detected in sesame flavor-type Baijiu, and the detected content was 18.78 ± 0.34 μg/L [14]. A tripeptide, Pro-His-Pro (PHP), was identified in Guojing and Gujinggong Baijiu with a content of 66.524 ± 5.769 μg/L [15]. Also, the antioxidant activity of Ala-Lys-Arg-Ala (AKRA) isolated from sesame flavor-type Baijiu was proved with a content of 8.497 ± 0.753 μg/L [16]. However, there are few reports on the oligopeptides in Baijiu due to their minimal concentration. The quality of Baijiu could be improved by controlling the concentration of various oligopeptides [17]. Therefore, to enrich oligopeptides in sauce-aroma Baijiu, we presented a novel distillation process that involved mixing Baijiu fermented grains with soy sauce residue to increase the oligopeptide concentration. The concentration of oligopeptides was increased to 0.912 mg/mL.

Soy sauce is a very well-known traditional fermented food in China. It was found that there were 237 taste-related dipeptides and 5 taste-related dipeptides in soy sauce [18]. Additionally, ACE-inhibitory dipeptides were identified in salt-free soy sauce, including Ala-Phe and Ile-Phe [19]. Soy sauce residue (Jiangzha in Chinese), produced by pressing, is a cheap and protein-rich biological resource [20]. However, the application value of soy sauce residue is limited because of its high salt content, poor palatability, and difficult storage. This study aims to separate and identify antioxidant oligopeptides from the mixed distillate of Baijiu fermented grains of sauce-aroma Baijiu (Jiupei) and soy sauce residue (Jiangzha) and explore their scavenging mechanisms for free radicals. It provides the preliminary basis for the high-value utilization of soy sauce residue and the development of antioxidant oligopeptides in sauce-aroma Baijiu.

## 2. Material and Methods

### 2.1. Materials

#### 2.1.1. Chemicals

Chemical reagents, including absolute ethanol, formic acid, and acetonitrile, were purchased from Beijing Soleibao Co., Ltd. (Beijing, China). GSH, sodium dihydrogen phosphate, 2,2-diphenyl-1-picrylhydrazyl (DPPH), 2,2′-azino-bis (3-ethylbenzothiazoline-6-sulfonic acid) (ABTS), potassium persulfate, ethylenediaminetetraacetic acid (EDTA), and microporous adsorption resin XAD-16 were from Shanghai McLean Reagent Co., Ltd. (Shanghai, China). Phenanthroline, ferrous sulfate, and hydrogen peroxide were purchased from Sinopharmaceutical Group Chemical Reagent Co., Ltd. (Beijing, China).

#### 2.1.2. Preparation of Mixed Distillation of Jiupei and Jiangzha

Jiupei was the fermented grains of sauce-aroma Baijiu from the eighth-round fermentation, and tail wine was the waste liquor from the seventh-round distillation of sauce-aroma Baijiu, which was collected from a brewing workshop of Longjiang Baijiu in Hunan Xiangjiao Co., Ltd. (Shaoyang, China). Jiangzha was the residue obtained from the pressing process of soy sauce at Hunan Longtan Soy Sauce Co., Ltd. (Xiangtan, China). The mixture of 50 g Jiupei and 50 g Jiangzha was added to a bottom flask, followed by an addition of 200 mL of the tail wine. The mixture was then distilled at 0.095 MPa with a temperature lower than 15 °C using a vacuum pump (Kenji Science and Technology Development Co., Ltd., Changsha, China). After distillation for 55 min at 70 °C, the capacity bottle was removed, sealed, and cooled to room temperature. The mixed distillate of Jiupei and Jiangzha was obtained and named MDFS.

### 2.2. Structural Identification of the Peptides

MDFS was dissolved in 95% liquid A (0.1% formic acid aqueous solution) containing 5% liquid B (84% acetonitrile in an aqueous solution containing 0.1% formic acid) and fed to the Zorbax 300SB-C18 column (Agilent Technologies, Wilmington, DE, USA) by the automatic injector equipped with liquid chromatography–mass spectrometry (LC–MS) system (Thermo Fisher, Waltham, MA, USA). The relevant gradient condition was set as follows: 0–50 min, liquid B linear gradient from 4% to 50%; 50–54 min, liquid B linear gradient from 50% to 100%; 54–60 min, liquid B maintained at 100%. Mass spectrometry was performed by a Q Exactive mass spectrometer (Thermo Fisher, MA, USA). The analysis time was 60 min, and the detection of positive ions was performed. The mass-charge ratio of peptides and their fragments was collected according to the following methods: 10 fragment profiles (MS2 scan) were collected after each full scan, and the original documents were tested by mass spectrometry (Raw File). Mass spectrums were analyzed using MaxQuant 1.5.5.1 software to retrieve the self-built database, which includes information on the raw material (barley, sorghum, and soybean), fungus (*Aspergillus*, *Rhizopus*, *Penicillium*, and *Alternaria*), yeast, and bacteria (lactic acid bacteria, acetic acid bacteria, and butyric acid bacteria) [21].

### 2.3. Synthesis of Peptides

According to the confidence level (>8.00), relative peak intensity (>500,000), and PeptideRanker score (>0.60), the representative oligopeptides were selected. The synthesis of the oligopeptides was entrusted to Cellmano Biotechnology Company (Hefei, China) according to their amino acid sequence for follow-up research.

### 2.4. Determination of the Antioxidant Activity of the Peptides

#### 2.4.1. DPPH Radical Scavenging Rate

DPPH radical scavenging capacity was evaluated through a method proposed by Shimada et al. [22] with slight modifications. The sample (500 µL) was mixed with 500 µL of 0.2 mmol/L DPPH in 95% ethanol and kept for 30 min at room temperature in the dark. The absorbance of the resulting solution was measured at 517 nm. All data were expressed as means ± standard deviations (SD) of triplicate analyses. The DPPH radical scavenging rate was calculated as follows:

(1) $$DPPH\;radical\;scavenging\;rate\;(\%)=\left(1-\frac{{Abs}_{sample}-{Abs}_{blank}}{{Abs}_{control}-{Abs}_{blank}}\right)\times 100$$

where Abs_sample_ is the absorbance of the sample in the presence of DPPH•; Abs_control_ is the absorbance of 95% ethanol (without peptides) in the presence of DPPH•; Abs_blank_ is the absorbance of the sample in the absence of DPPH•. GSH was used as the positive control.

#### 2.4.2. ABTS•^+^ Scavenging Rate

According to the method reported by Mejri et al. [23], with modifications, an ABTS•^+^ stock solution containing 7 mmol/L ABTS and 2.45 mmol/L potassium persulfate was prepared and kept in the dark for 14 h. The working solution was prepared by mixing the ABTS•^+^ stock solution with potassium persulfate (0.5 mmol/L, pH 7.4) to reach an absorbance of 0.70 ± 0.05 at 734 nm. A total of 200 µL of the sample was mixed with 3 mL of ABTS•^+^ working solution for 6 min in the dark. Then, the absorbance was measured at 734 nm. All data were expressed as means ± standard deviations (SD) of triplicate analyses. The ABTS•^+^ scavenging rate was calculated using the following equation:

(2) $$ABTS\;radical\;scavenging\;rate\;(\%)=\frac{{Abs}_{0}-{Abs}_{S}}{{Abs}_{0}}\times 100$$

where Abs_s_ is the absorbance of 200 µL of sample with 3 mL of ABTS•^+^ working solution, and Abs_0_ is the absorbance of 200 µL of buffer with 3 mL of ABTS•^+^ working solution. GSH was employed as the positive control.

#### 2.4.3. Hydroxyl Radical Scavenging Rate

According to the Li et al. [24] method with minor modifications, hydroxyl radicals were generated by the reaction of hydrogen peroxide with Fe (II), which was monitored by its reaction with phenanthroline. Antioxidant compounds inhibit the oxidation of Fe (II) to Fe (III) and the formation of •OH. A total of 0.6 mL of 5 mmol/L phenanthroline solution with 0.4 mL of potassium persulfate (0.2 mol/L, pH 7.4), 0.6 mL of 5 mmol/L ferrous sulfate, 0.6 mL of sample, 0.6 mL of EDTA (15 mmol/L), and 0.8 mL of 0.1% (*v*/*v*) hydrogen peroxide were mixed. The mixture was incubated for 1 h at 37 °C, and the absorbance was measured at 536 nm. All data were expressed as means ± standard deviations (SD) of triplicate analyses. The hydroxyl radical scavenging rate was calculated according to the following equation:

(3) $$Hydroxyl\;radical\;scavenging\;rate\left(\%\right)=\frac{A_{sample}-A_{damaged}}{A_{undamaged}-A_{damaged}}\times 100$$

where Abs_sample_ is the absorbance of the sample, A_undamaged_ is the absorbance of the buffer without hydrogen peroxide, and A_damaged_ is the absorbance of the buffer without peptide. GSH was employed as the positive control.

### 2.5. IC_50_ Calculation

The peptide solutions with five concentration gradients of 0.1, 0.2, 0.4, 0.8, and 1.6 mg/mL were prepared, and the corresponding DPPH•, ABTS•^+^, and hydroxyl radical scavenging rates were measured in accordance with the above-mentioned methods. The corresponding peptide concentration (IC_50_) value was calculated when the free radical scavenging rate was 50% by GraphPad Prism.

### 2.6. Molecular Docking Simulation

Molecular docking was performed according to our previous study with slight modifications [25]. In brief, the 3D structures of the free radicals were obtained by ChemBioDraw (OriginLab Co., CambridgeSoft, Cambridge, UK), converted into PDB files, and further utilized to generate the 3D structure of the peptides using an online tool (https://www.novopro.cn/tools/smiles2pdb.html, accessed on 16 January 2024). The molecular docking simulations between the peptides and the odor substances were performed using the AutoDock4 software, employing the AutoGrid and AutoDock modules. Subsequently, visual analysis was conducted using Ligplot v.2.2.8 software (https://www.ebi.ac.uk/thornton-srv/software/LigPlus/, accessed on 10 December 2023) [26].

### 2.7. Statistical Analysis

The data for the peptide identification were processed using Excel 2019. The results of the bioassay of oligopeptide were analyzed using IBM SPSS Statistics 26 for significant difference testing. All data were expressed as means ± standard deviations (SD) of triplicate analyses. *p* < 0.05 was considered to be statistically significant, and values with superscript letters a, b, c, d, and e were significantly different across columns (*p* < 0.05).

## 3. Results and Discussion

### 3.1. Structural Characterization of the Peptides from Mixed Distillation of Jiupei and Jiangzha

**Figure A1:**
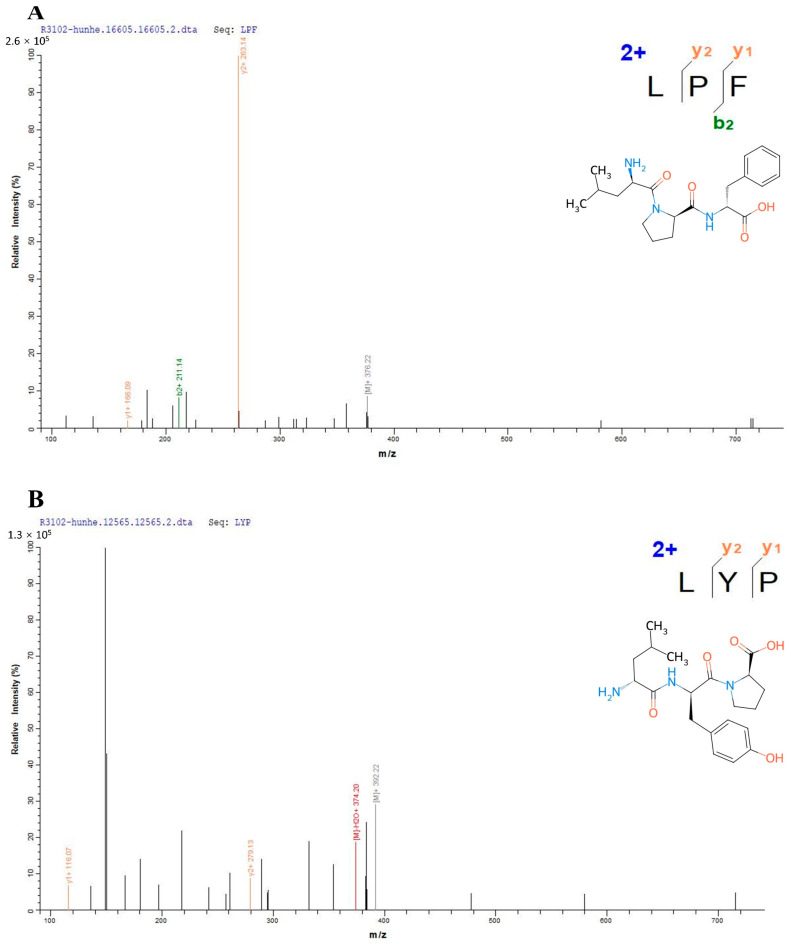

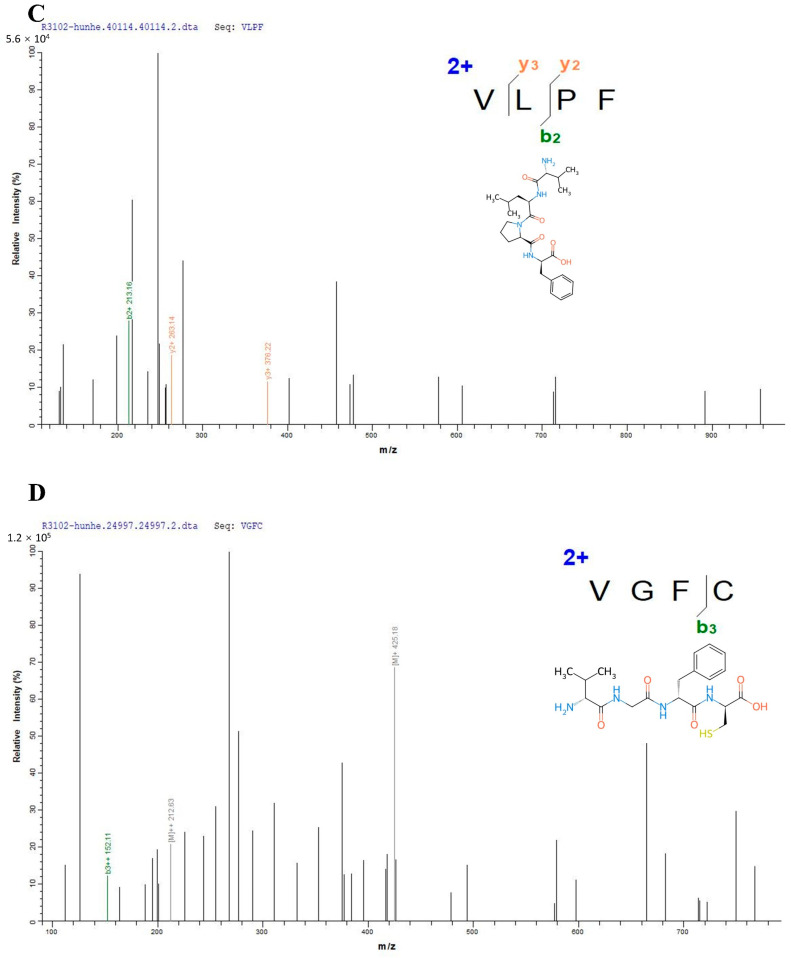

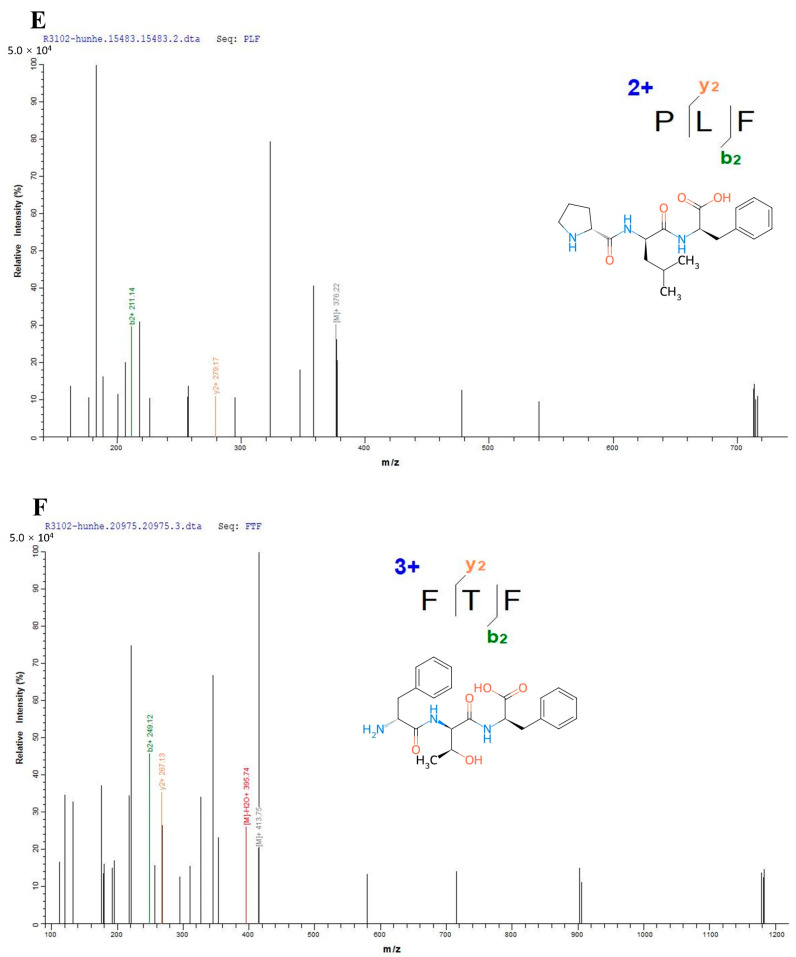
Identification of the peptides in the mixed distillation of Jiupei and Jiangzha by LC–MS. (**A**) LPF; (**B**) LYP; (**C**) VLPF; (**D**) VGFC; (**E**) PLF; (**F**) FTF.

According to the oligopeptides found in the MDFS, the confidence score (>8.00), relative peak intensity (>500,000), and PeptideRanker score (>0.60), the representative sequences with high reliability were selected from the oligopeptides identified in this study. Six oligopeptides (Figure A1) were obtained, including Leu-Tyr-Pro (LYP; confidence score: 15.73; intensity: 957,740), Phe-Thr-Phe (FTF; confidence score: 29.324; intensity: 490,250), Leu-Pro-Phe (LPF; confidence score: 20.41; intensity: 552,360), Val-Leu-Pro-Phe (VLPF; confidence score: 24.24; intensity: 9,846,800), Val-Gly-Phe-Cys (VGFC; confidence score: 8.22; intensity: 1,878,800), and Pro-Leu-Phe (PLF; confidence score: 36.05; intensity: 6,370,900).

Oligopeptides are generally protonated under mass spectrometer conditions. As other chemical bonds on the side chain are difficult to break at low energy, cleavage mainly occurs on the amide bond. b ion and y ion are the main fragment ions when the collision energy is lower than 200 eV [25]. From the identification results, most of the oligopeptides in the MDFS were tripeptides and tetrapeptides, which was consistent with the previous reports of peptides in Baijiu [17]. The biological activity of oligopeptides mainly depends on their structural characteristics, such as chain length, amino acid composition, hydrophobicity, and charge distribution [27]. Additionally, antioxidant oligopeptides generally contain 0–20 amino acids; their sequences containing Pro, Gly, Ala, and Val have potential antioxidant activity; oligopeptides with aromatic rings (Tyr, Trp, Phe, and His) and sulfur groups (Met and Cys) directly quench free radicals [28]. Therefore, LPF, LYP, VLPF, VGFC, PLF, and FTF might have antioxidant activity.

### 3.2. Antioxidant Activity of the Peptides from the Mixed Distillation of Jiupei and Jiangzha

As known in Table 1, the antioxidant activities of LPF, LYP, VLPF, VGFC, PLF, and FTF were different. The DPPH radical scavenging capacity (IC_50_) of VGFC was 0.51 mg/mL, which was similar to that of GSH’s DPPH radical scavenging capacity. Secondly, FTF had a relatively higher scavenging capacity for DPPH radical (1.32 mg/mL). The other oligopeptides (LPF, LYP, VLPF, and PLF) have the weak scavenging capacity of the DPPH radical. Furthermore, the ABTS•^+^ radical scavenging capacity of VLPF and VGFC was significantly higher than that of other oligopeptides, close to GSH. These oligopeptides showed ideal hydroxyl radical scavenging capacity, among which VGFC had the strongest capacity, and their IC_50_ was 0.89 mg/mL, which was similar to GSH. In terms of amino acid composition, glycine (Gly) plays an important role in the free radical scavenging activity of antioxidant peptides. It shows strong antioxidant activity by transferring electrons to free radicals, and its fat side chain can enhance the solubility of antioxidant peptides in oil, thus delaying the oxidation of oil [29]. Thus, VGFC may be able to pass through the cell membrane more effectively to scavenge free radicals, block or terminate intracellular free radical chain reactions, and thus protect cells from damage [30]. Guo et al. identified antioxidant peptides in Guangdong glutinous rice wine, including VLSGA (EC50: 0.118 mg/mL), VISGA (EC50: 0.056 mg/mL), MGKAA (EC50: 0.054 mg/mL), and GHVAA (EC50: 0.027 mg/mL) [31].

In particular, VGFC had the strongest antioxidant activity, and its effect was close to GSH. GSH is a recognized antioxidant peptide that could inhibit the oxidative deterioration of food and prevent oxidative stress and injury [32,33]. VGFC was of great research value and could be widely used as antioxidants or nutritional supplements in functional foods. In this study, these oligopeptides had low molecular weights with a range from 375.21 Da to 474.28 Da. The result was similar to the report that the peptides below 5 kDa have a strong scavenging capacity for DPPH radicals [23]. Moreover, these oligopeptides had hydrophobic amino acids located at the N-terminal, which were related to the antioxidant activity of oligopeptides because those hydrophobic amino acids can interact more easily with free radicals, promoting oligopeptides’ full bind with free radicals and scavenging free radicals better [34,35].

### 3.3. Intermolecular Interaction between the Peptides and Free Radicals

#### 3.3.1. The Peptides and DPPH Free Radicals

**Figure 1:**
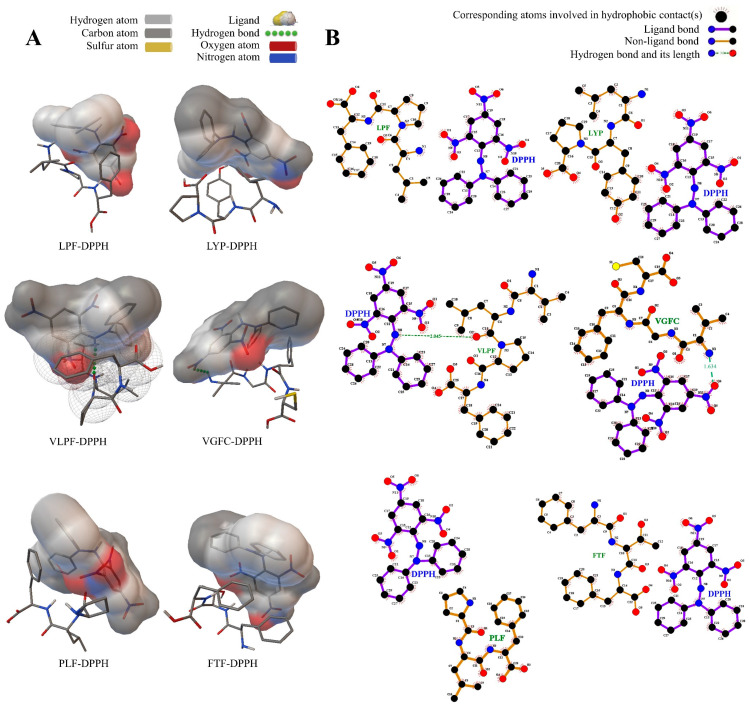
Computational visualization of the molecular docking between the peptides and the DPPH free radical. (**A**) The 3D mode of the peptides and the DPPH free radical; (**B**) the interaction force model of the peptides and the DPPH free radical.

In Table 2, LPF, LYP, VLPF, VGFC, PLF, and FTF to DPPH radicals all had intermolecular interaction, showing stronger binding energy. Oligopeptides can bind stably with DPPH radicals. For DPPH radicals, the binding energies of LPF, LYP, VLPF, VGFC, PLF, and FTF were 1.88 kcal/mol, 6.15 kcal/mol, 4.96 kcal/mol, −1.26 kcal/mol, 14.86 kcal/mol, and −0.97 kcal/mol, respectively. The binding energy of VGFC to DPPH radicals was the most stable, followed by FTF. Previous research has demonstrated that ligands interact with receptors through various intermolecular forces, such as hydrophobic force, van der Waals force, hydrogen bond, π bond, and electrostatic interaction. Among these forces, hydrogen bond interaction is considered to be particularly strong [36]. In Figure 1A, VGFC and DPPH• formed a hydrogen bond with a distance of 1.634 Å, and the electrostatic energy contributed greatly to the interaction between VGFC and DPPH radicals. Consequently, VGFC was more likely to interact with DPPH free radicals, which was similar to the results of the determination of the antioxidant activity of oligopeptides. Zheng et al. identified SSLFR, QFTPL, and FTYPR from rice wine as having antioxidant activities. These peptides can effectively interact with DPPH radical molecules, resulting in good DPPH radical scavenging activity [37]. Moreover, hydrogen atom transfer (HAT) is believed to be the main mechanism of DPPH scavenging [38]. Amino acid residues such as Tyr, Ala, and Pro, which are effective hydrogen donors, have been reported to have a significant role in the antioxidant capacity of peptides [39]. As known in Figure 1B, DPPH radicals tended to form hydrogen bonds with Leu and Val, which suggested that oligopeptides containing Leu and Val may have better affinity with DPPH radicals so that oligopeptides could exert their antioxidant activity better.

#### 3.3.2. The Peptides and ABTS^+^ Free Radicals

**Figure 2:**
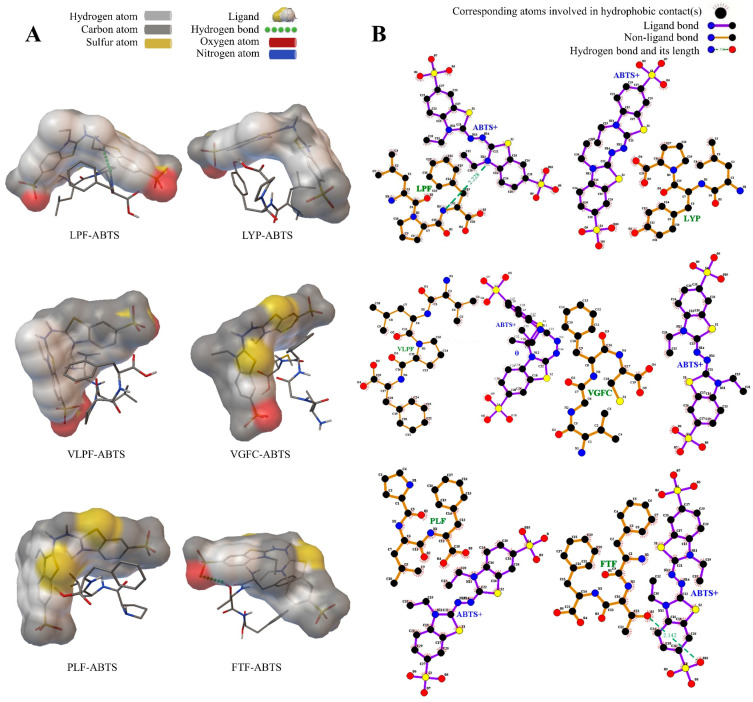
Computational visualization of the molecular docking between the peptides and the ABTS^+^ free radical. (**A**) The 3D mode of the peptides and the ABTS^+^ free radical; (**B**) the interaction force model of the peptides and the ABTS^+^ free radical.

The binding energies of LPF, LYP, VLPF, VGFC, PLF, and FTF to ABTS•^+^ radicals were 61.63 kcal/mol, 36.91 kcal/mol, 29.18 kcal/mol, −1.33 kcal/mol, 20.83 kcal/mol, and 62 kcal/mol in Table 2. The binding energy of VGFC was the strongest, suggesting that VGFC was more likely to interact with ABTS•^+^ radicals, thus scavenging them. Moreover, the binding energy of PLF was 20.83 kcal/mol, and the electrostatic binding energy was 0.43 kcal/mol. As known in Figure 2A, Thr (H), the amino acid residue of FTF, formed a hydrogen bond with the ABTS•^+^ radicals with a hydrogen bond distance of 2.142 Å. The amino acid residue Phe (HN) of LPF provided hydrogen atoms and formed a hydrogen bond with the oxygen atoms of the ABTS•^+^, which indicates that the ABTS•^+^ were more likely to form a hydrogen bond with Phe and Thr. Additionally, antioxidant peptides can scavenge reactive oxygen species (ROS) directly by donating hydrogen or electrons. The hydrophobic interactions and the presence of hydrogen bonds are closely linked to the capacity of active peptides to scavenge free radicals [40]. In our study, the HN^−^ functional group usually forms a hydrogen bond with the ligand oxygen atom in the intermolecular interaction (Figure 2B). This could be because of the higher electronegativity of the HN^−^ functional group and the oligopeptide molecular conformation.

#### 3.3.3. The Peptides and Hydroxide Free Radicals

**Figure 3:**
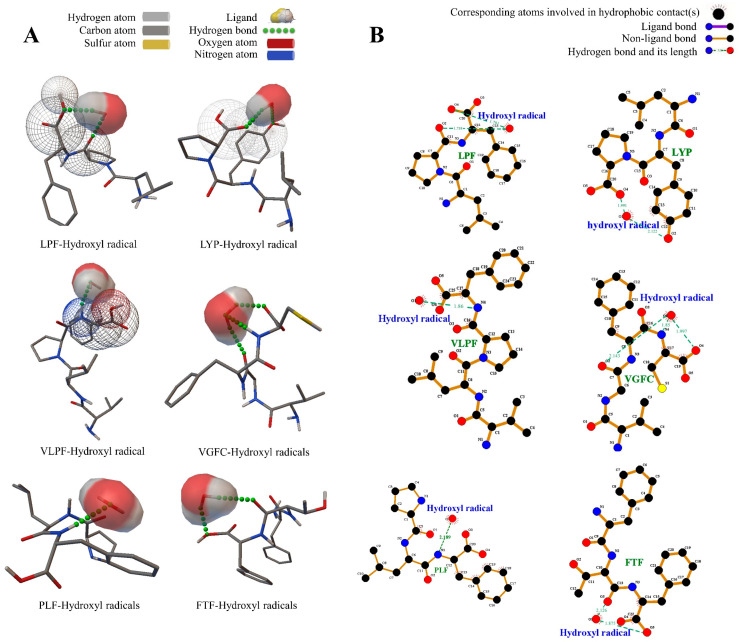
Computational visualization of the molecular docking between the peptides and the hydroxy free radical. (**A**) The 3D mode of the peptides and the hydroxy free radical; (**B**) the interaction force model of the peptides and the hydroxy free radical.

The binding energy of VGFC to hydroxide-free radicals was also the strongest, and the binding energy was −1.93 kcal/mol. Moreover, three hydrogen bonds were formed between amino acid residues Cys (O), Gly (O), and Cys (HN) of VGFC and hydroxide radicals, and the hydrogen bond distances were 1.997 Å, 2.143 Å, and 1.85 Å (Figure 3), suggesting that VGFC has a stronger ability to clear hydroxide free radicals. On the whole, the interaction between VGFC and DPPH radicals, ABTS•^+^ radicals, and hydroxide radicals was the most stable, which also confirmed the results of antioxidant activity determination. Additionally, it was found that oligopeptides with Leu, Val, Phe, Pro, and Cys were more likely to form hydrogen bonds with DPPH radicals, ABTS•^+^ radicals, and hydroxide radicals. Specifically, hydrophobic amino acids such as Leu, Val, Phe, and Pro were also reported to play an important role in scavenging radicals and are conducive to antioxidant activity [41]. Consequently, the oligopeptides with Leu, Val, Phe, and Pro were likely to be close to each other by forming hydrogen bonds with radicals and then scavenging them and exerting antioxidant activity.

## 4. Conclusions

The structure of oligopeptides from MDFS was identified by LC–MS, and we explored the intermolecular mechanism of scavenging DPPH•, ABTS•^+^, and hydroxide radicals. The potential antioxidant capacity of VGFC was the strongest, and its scavenging capacity for DPPH•, ABTS•^+,^ and hydroxide radicals (IC_50_) was 0.51 mg/mL, 0.53 mg/mL, and 0.89 mg/mL, respectively, which were similar to GSH. The results of molecular docking suggested that VGFC formed stable hydrogen bonds with these radicals and thus showed stronger potential antioxidant capacity. Additionally, the oligopeptides composed of Leu, Val, Phe, and Pro showed a high affinity for hydrogen bonding with free radicals, so they can better scavenge radicals and exert antioxidant capacity. It provided a new idea for the development and utilization of bioactive oligopeptides in sauce-aroma Baijiu. However, the effects of these oligopeptides on oxidative stress, the activities of catalase and superoxide dismutase in cells, and the mechanism of the antioxidant activity of oligopeptides require further investigation.

## Figures and Tables

**Table 1:** Antioxidant activity of the peptides from the mixed distillation of Jiupei and Jiangzha.

Peptide	Radical Scavenging Ability (IC_50_, mg/mL)		
	DPPH•	ABTS•^+^	Hydroxyl•
Control (GSH)	0.51 ± 0.10 ^a^	0.52 ± 0.10 ^a^	0.81 ± 0.36 ^a^
LPF	2.68 ± 1.78 ^c^	3.90 ± 2.72 ^c^	4.05 ± 0.37 ^c^
LYP	5.91 ± 3.08 ^e^	8.56 ± 2.50 ^d^	3.85 ± 0.37 ^c^
VLPF	3.94 ± 1.35 ^d^	0.53 ± 0.05 ^a^	5.45 ± 0.57 ^e^
VGFC	0.51 ± 0.01 ^a^	0.53 ± 0.11 ^a^	0.89 ± 0.18 ^b^
PLF	9.48 ± 3.15 ^e^	8.50 ± 0.19 ^d^	3.99 ± 0.10 ^c^
FTF	1.32 ± 2.45 ^b^	3.17 ± 0.33 ^b^	4.41 ± 0.10 ^d^

Note: GSH, glutathione; values with superscript letters a–e are significantly different across columns (*p* < 0.05).

**Table 2:** Molecular docking between the peptides and the free radicals.

Sequence	Radical	Electrostatic Energy (kcal/mol)	Binding Energy (kcal/mol)	Number of Hydrogen Bonds	Hydrogen Bond Distance	Residues Formed Hydrogen Bonds with the Ligand
LPF	DPPH	−0.16	1.88	0	-	-
	ABTS	0.75	61.63	1	2.228	Phe (HN)
	Hydroxyl	−0.34	−1.48	2	1.738, 1.781	Phe (O), Pro (O)
LYP	DPPH	−0.59	6.15	0	-	-
	ABTS	0.57	36.91	0	-	-
	Hydroxyl	−0.22	−1.46	2	1.991, 2.122	Pro (O), Tyr (O)
VLPF	DPPH	0.22	4.96	1	2.045	Leu (O)
	ABTS	0.62	29.18	0	-	-
	Hydroxyl	−0.24	−1.78	1	1.86	Phe (HN)
VGFC	DPPH	−1.29	−1.26	1	1.634	Val (HN)
	ABTS	0.03	−1.33	0	-	-
	Hydroxyl	−0.26	−1.93	3	1.997, 2.143, 1.85	Cys (O), Gly (O), Cys (HN)
PLF	DPPH	−1.09	14.86	0	-	-
	ABTS	0.43	20.83	0	-	-
	Hydroxyl	−0.02	−1.75	1	2.189	Phe (HN)
FTF	DPPH	−0.8	−0.97	0	-	-
	ABTS	−0.23	62	1	2.142	Thr (H)
	Hydroxyl	−0.3	−1.47	2	1.875, 2.126	Phe (O), Thr (O)

## Data Availability

The data presented in this study are available on request from the corresponding author. The data are not publicly available due to institutional policies regarding data protection.
